# Nr4a2 Transcription Factor in Hippocampal Synaptic Plasticity, Memory and Cognitive Dysfunction: A Perspective Review

**DOI:** 10.3389/fnmol.2021.786226

**Published:** 2021-11-22

**Authors:** Judit Català-Solsona, Alfredo J. Miñano-Molina, José Rodríguez-Álvarez

**Affiliations:** ^1^Departament de Bioquímica i Biologia Molecular, Institut de Neurociències, Universitat Autònoma de Barcelona, Cerdanyola del Vallès, Spain; ^2^Centro de Investigación Biomédica en Red Sobre Enfermedades Neurodegenerativas (CIBERNED), Madrid, Spain; ^3^Dominick P. Purpura Department of Neuroscience, Albert Einstein College of Medicine, New York, NY, United States

**Keywords:** Nr4a2, transcription factor, hippocampus, synaptic plasticity, brain, learning, memory

## Abstract

Long-lasting changes of synaptic efficacy are largely mediated by activity-induced gene transcription and are essential for neuronal plasticity and memory. In this scenario, transcription factors have emerged as pivotal players underlying synaptic plasticity and the modification of neural networks required for memory formation and consolidation. Hippocampal synaptic dysfunction is widely accepted to underlie the cognitive decline observed in some neurodegenerative disorders including Alzheimer’s disease. Therefore, understanding the molecular pathways regulating gene expression profiles may help to identify new synaptic therapeutic targets. The nuclear receptor 4A subfamily (Nr4a) of transcription factors has been involved in a variety of physiological processes within the hippocampus, ranging from inflammation to neuroprotection. Recent studies have also pointed out a role for the activity-dependent nuclear receptor subfamily 4, group A, member 2 (Nr4a2/Nurr1) in hippocampal synaptic plasticity and cognitive functions, although the underlying molecular mechanisms are still poorly understood. In this review, we highlight the specific effects of Nr4a2 in hippocampal synaptic plasticity and memory formation and we discuss whether the dysregulation of this transcription factor could contribute to hippocampal synaptic dysfunction, altogether suggesting the possibility that Nr4a2 may emerge as a novel synaptic therapeutic target in brain pathologies associated to cognitive dysfunctions.

## Introduction

Nuclear receptor subfamily 4, group A (Nr4a), which belongs to the nuclear receptor superfamily, consists in a family of three close-related immediate early genes that encode three orphan nuclear receptors (*Nr4a1/NGFI-B*, *Nr4a2/Nurr1*, and *Nr4a3/NOR-1*). They are expressed in a wide variety of metabolically demanding and energy dependent tissues, such as skeletal muscle, adipose tissue, heart, kidney, T-cells, liver and distinct but overlapping regions of the brain (Zetterström et al., 1996). The three members exhibit tissue-specific expression and hence their roles are context as well as tissue-specific.

Nr4a proteins function as transcription factors that recognize DNA response elements to regulate the expression of a variety of genes involved in multiple biological processes including proliferation, metabolism, immunity, cellular stress, apoptosis, DNA repair, and angiogenesis (Safe et al., 2016). Nr4a activity depends not only on its transcriptional levels (including miRNA targeting) but also on alternative splicing, post-translational modifications, subcellular localization and interaction with other nuclear receptors (Maxwell and Muscat, 2006). These transcriptional regulators may be stably expressed or induced as immediate early genes in response to a wide range of physiological signals, including synaptic activity (Pegoraro et al., 2010). The ability to sense and rapidly respond to changes in the cellular environment appears to be a hallmark of this subfamily of orphan nuclear receptors.

In peripheral tissues, these receptors have been involved in the onset and progression of various types of cancer, inflammation, atherosclerosis and obesity (Ranhotra, 2015). Notably, Nr4a transcription factors have also emerged as essential mediators in several physio-pathological situations in the central nervous system (CNS). The Nr4a family of transcriptional regulators has been reported to modulate hippocampal synaptic plasticity and hippocampal-dependent memory (Hawk and Abel, 2011; Bridi and Abel, 2013) and the alteration of expression or function of these factors has been associated to neurodegenerative diseases (Skerrett et al., 2014), intellectual disability such as autism (Chuang et al., 2015), schizophrenia (Rojas et al., 2007; Ruiz-Sánchez et al., 2021) and epilepsy (Zhang et al., 2016).

This review will primarily focus on the current knowledge about Nr4a2 contribution to the active-dependent processes that underlie synaptic plasticity, learning and memory and the evidence supporting a critical role of this factor in several brain pathologies associated to cognitive dysfunction.

## Nr4a2 Transcription Factor

Nuclear receptor subfamily 4, group A, member 2 (Nr4a2), which belongs to the Nr4a subfamily of transcription factors, is primarily expressed in neurons of diverse areas of the CNS, particularly in the substantia nigra pars compacta, ventral tegmental area and limbic area (Zetterström et al., 1996). Moreover, it is also expressed in the hippocampus, subiculum, temporal cortex, olfactory bulb, cerebellum, posterior hypothalamus, and habenuclear nuclei (Saucedo-Cardenas and Conneely, 1996; Quina et al., 2009). Nevertheless, *Nr4a2* is expressed not only in neurons, but also in non-neuronal cells such as microglia, astrocytes or endothelial cells (Fan et al., 2009; Saijo et al., 2009) and it is found not only in the CNS but also in other tissues, including the bone, synovial tissues, adrenal gland or the intestine.

**FIGURE 1:**
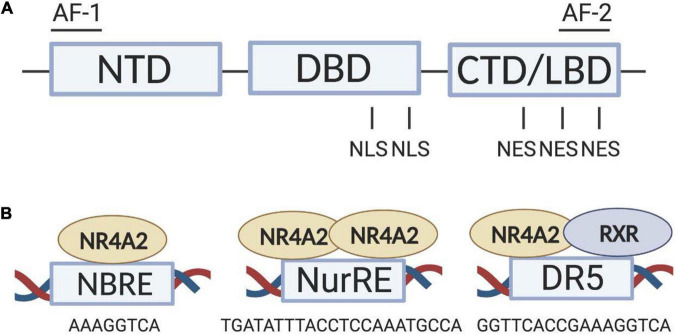
Nr4a2 structure and binding to target genes. **(A)** Structural domains of Nr4a2, which include NTD, DBD and LBD. Modulator domains (AF-1 and AF-2) and localization signals (NLS and NES) are also depicted. **(B)** Nr4a2 DNA-binding sites as monomers at NBRE sites, as dimers (homodimers or heterodimers, not shown) at NurRE sites and as heterodimers with RXR at DR5 sites. AF, activation function; CTD, C-terminal domain; DBD, DNA-binding domain; LBD, ligand-binding domain, NBRE, nerve growth factor-inducible-β-binding response element; NES, nuclear export signal; NLS, nuclear localization signal, NTD, N-terminal domain; NurRE, nur-response element; RXR, retinoic X receptor.

Nr4a2 structure (Figure 1A) is composed by a modulator domain, referred to as the activation function (AF-1) or the N-terminal domain (NTD), a conserve DNA-binding domain (DBD) and a ligand-binding domain (LBD) containing its transactivation-dependent AF-2 in the C-terminal domain (CTD) (Ichinose et al., 1999). Classically, nuclear receptor activation is accomplished by the binding of a lipophilic ligand in a hydrophobic pocket within the LBD. By contrast, the crystal structure of Nr4a2 reveals that the Nr4a2-LBD lacks a classical biding pocket for coactivators and/or ligands because of the tight packing of side chains from several bulky hydrophobic side-chain residues, adopting a canonical protein fold resembling that of an agonist-bound, transcriptionally active LBD (Wang et al., 2003). Thus, it is believed that a major difference between Nr4a2 and classical nuclear receptors is the absence of endogenous ligand regulation. Therefore, Nr4a2 activity will be regulated at the level of gene expression and protein stability (Maxwell and Muscat, 2006). Nevertheless, during the last years, several studies have identified synthetic ligands, known as Nr4a2 agonists, which can modulate Nr4a2 transcriptional function (Hammond et al., 2015; Kim et al., 2015; Chatterjee et al., 2020). Identification of such Nr4a2 agonists triggered the possibility of a pharmacological therapeutic approach for diseases related to Nr4a2 (Skerrett et al., 2014; Kim et al., 2015; Moon et al., 2019; Chatterjee et al., 2020; Jeon et al., 2020). The canonical view of Nr4a2 as an orphan nuclear receptor has been recently challenged by some studies showing that the Nr4a2-LBD is highly dynamic with solvent accessibility and the capability to expand (de Vera et al., 2019) allowing the binding of putative endogenous ligands (Bruning et al., 2019; Rajan et al., 2020).

Nr4a2 protein regulates both positively and negatively the transcription of its target genes by directly binding to response elements in their promoters. Two zinc fingers of the highly conserved Nr4a2-DBD as a monomer or homodimer can bind the nerve growth factor-inducible-β-binding response element (NBRE; 5′-AAAGGTCA-3′) or as homodimer or heterodimer with Nr4a1 can attach to the Nur response element (NurRE; 5′-TGACCTTT-N6-AAAGGTCA-3′). Moreover, as monomer, homodimer or heterodimer, Nr4a2 can dimerize with the retinoic X receptor (RXR) and bind to a motif referred to as DR5 (Figure 1B). This union is permissive; meaning that ligand binding of RXR typically causes full activation of the entire heterodimer (Jiang et al., 2019).

Nr4a2 is known to be a key regulator of the midbrain dopaminergic neurons differentiation, maintenance and survival (Saucedo-Cardenas et al., 1998; Sousa et al., 2007; Jo et al., 2009), being responsible for the transcription of several genes involved in the dopaminergic neuronal phenotype, ranging from genes regulating dopaminergic metabolism, differentiation and neurotransmission (tyrosine hydroxylase –*TH*–, vesicular amine transporter 2 –*VMAT*–, dopamine transporter –*DAT*–), mitochondrial function (sodium oxide dismutase 1 –*SOD1*–, Ts translation elongation factor mitochondrial –*TSFM*–, cyclo-oxigenase 5β –*COX5*β–) and dopaminergic neuronal survival (*Ret*) (Kadkhodaei et al., 2009; Decressac et al., 2013). In dopaminergic neurons, Nr4a2 also has an essential role in the neuroprotection and anti-inflammatory responses after exposure to neuropathological stress or insults. It has been reported that *Nr4a2*-null heterozygous dopaminergic neurons exhibit greater vulnerability to neurotoxins (Le et al., 1999). Importantly, Nr4a2 protects dopaminergic neurons from neuroinflammation insults not only through its function in neurons, but also glial cells (Bensinger and Tontonoz, 2009), since it is able to suppress inflammatory gene expression in microglia and astrocytes through transcriptional repression of the NFκB transcription factor, essential to induce inflammatory responses (Saijo et al., 2009).

*Nr4a2* contains a “half-CRE” site in its promoter, being controlled by the CREB signaling pathway in many systems, including the hippocampus, which is a cascade critical for the transcription of diverse plasticity- and memory-related genes (Volakakis et al., 2010; Barneda-Zahonero et al., 2012). Although CREB binds with higher affinity to the full 8-base consensus CRE site, it also binds to several variations of this sequence, including the so-called “half-CRE” site (5′CGTCA-3′ and 5′-TGACG-3′), which may be found in many promoters of CREB target genes including *Nr4a2* (Zhang et al., 2005). CREB has been extensively studied in the brain since it is involved in a variety of relevant processes such as learning and memory or neuronal survival, among others (Benito and Barco, 2015). For example, it is known that CREB activity is crucial for the maintenance of long-term plasticity and memory formation (Bourtchuladze et al., 1994; Bartsch et al., 1998; Kaldun and Sprecher, 2019). Moreover, the genetic program downstream CREB is complex and different transcriptional programs could be elicited depending on the triggering stimulus, cellular context, and upstream signaling events (Nonaka et al., 2014). Furthermore, selective gene transcription by CREB is highly modulated by the recruitment of specific coactivators such as the CREB binding protein (CBP/p300) or CREB regulated Transcription Coactivator (CRTC1 and CRTC2) (Altarejos and Montminy, 2011). Reports showing that CREB modulates transcription of *Nr4a2* (Volakakis et al., 2010; Barneda-Zahonero et al., 2012) has fostered the interest for studying the putative role of Nr4a2 in synaptic plasticity and learning and memory processes.

## Role of Nr4a2 in Hippocampal Synaptic Plasticity, Learning, and Memory

Several reports have highlighted a role for the Nr4a family of transcription factors in hippocampal synaptic plasticity and cognitive functions and there is an overwhelming amount of evidence showing that synaptic plasticity is a fundamental mechanism contributing to learning and memory storage (Martin et al., 2000; Abraham et al., 2019). Studies revealing the implication of the Nr4a family of transcription factors in hippocampal synaptic plasticity have supported a putative role of these factors in learning and memory formation (Hawk and Abel, 2011). Early studies showed an increase in Nr4a1 mRNA in CA1 after contextual fear conditioning during consolidation (von Hertzen and Giese, 2005; Keeley et al., 2006). Later, it was found that expression of a dominant-negative form of Nr4a in the mouse forebrain, including the hippocampus, impaired long-term hippocampus-dependent contextual fear memory without affecting short-term contextual fear memory or hippocampus-independent cued fear memory (Hawk et al., 2012). Furthermore, it was also observed that histone deacetylase (HDAC) inhibitors-mediated memory enhancement was accompanied by an increase in the expression of Nr4a family members meanwhile blocking Nr4a family expression in the hippocampus prevented HDAC inhibitors-mediated improvement of long-term memory (Hawk et al., 2012). Supporting the role of Nr4a transcription factors in hippocampal synaptic plasticity associated to learning and memory, a strongly impaired transcription-dependent hippocampal long-term potentiation (LTP) maintenance of Schaffer collateral to CA1 synapses was observed in hippocampal slices from Nr4a dominant negative mutant mice whereas no deficits in basal synaptic transmission were observed (Bridi and Abel, 2013).

Both hippocampal LTP and long-term memory are enhanced by the pharmacological inhibition or genetic ablation of HDAC enzymes, which increase histone acetylation in a CREB-dependent manner (Vecsey et al., 2007). Importantly, HDAC inhibition failed to enhance LTP in hippocampal slices from Nr4a dominant negative mutant mice, indicating that Nr4a transcription factors are necessary for mediating the effects of HDAC inhibition on synaptic plasticity (Bridi and Abel, 2013).

Although these results supported the importance of the Nr4a family members for hippocampal memory formation, it is still uncertain which are the specific role for each member: Nr4a1, Nr4a2, and Nr4a3. A differential role in learning for Nr4a1 and Nr4a2 was reported by McNulty and co-workers (McNulty et al., 2012). Using small interfering RNA (siRNA), they found that downregulation of Nr4a2 disrupted long-term memory in the object location and object recognition tasks whereas Nr4a1 seemed to be only required for object location memory. Bridi and co-workers reported an enhancement of LTP when hippocampal slices were incubated with Nr4a1 activators, para-phenyl substituted di-indolylmethane compounds or DIM-C (Bridi et al., 2017). DIM-C-pPhOCH_3_ or DIM-C-pPhBr enhanced LTP at the CA3–CA1 hippocampal synapses. This enhancement was absent when LTP was triggered in slices from dominant-negative Nr4a mice. Several studies have evaluated the role of Nr4a2 in memory acquisition and consolidation, reporting an increase in the hippocampal expression of Nr4a family members after learning of hippocampus-dependent tasks. A specific increase of Nr4a2 in the hippocampus was observed after different memory-inducing activities. Acquisition of a spatial food search task in adult rats produced an increase in the levels of Nr4a2 mRNA in CA1 and CA3 subregions of the dorsal hippocampus (Peña de Ortiz et al., 2000) and the injection of antisense oligodeoxynucleotides targeting *Nr4a2* in the CA3 hippocampal area impaired long-term memory and reversal learning in an appetitive spatial learning task (Colón-Cesario et al., 2006). Relatedly, contextual fear conditioning increased Nr4a2 mRNA and promoter acetylation in a CREB/CBP-dependent manner (Vecsey et al., 2007; Bridi et al., 2017; Oliveira et al., 2018). The role of Nr4a2 in other hippocampal-dependent behavioral tasks like passive avoidance was also reported when *Nr4a2* heterozygous mice showed reduced ability to form long-term emotional memories (Rojas et al., 2007). Also, deep brain stimulation in rats by intracranial self-stimulation treatment immediately after the acquisition session of a two-way active avoidance conditioning showed both increased Nr4a2 protein levels in the hippocampus and improved retention (Aldavert-Vera et al., 2013). Accordingly with the above-commented results observed with HDAC inhibitors, it was also reported that a Nr4a2-dependent enhancement of novel object recognition long-term memory was observed when *HDAC3* expression was deleted in the CA1 area of the mice dorsal hippocampus (McQuown et al., 2011).

Cognitive decline is also associated with aging and *Nr4a2* expression gradually decreases in the gerbil hippocampus with increasing age (Ahn et al., 2018). Furthermore, Nr4a2 has also been recently related to long-term memory impairments in aged animals (Kwapis et al., 2019). Whereas adult or aged rats with intact cognitive functions showed an increase in *Nr4a2* expression in the dorsal hippocampus after the object recognition memory training, Nr4a2 failed to be induced in the cognitively impaired aged rats. Learning-induced Nr4a2 was also impaired in the hippocampus of aging mice. Thus, a cross-species impairment of *Nr4a2* hippocampal expression is observed in mice and rats with cognitive deficits. Interestingly, failed induction of Nr4a2 expression is reversed when *HDAC3* is deleted in the dorsal hippocampus. Deletion of *HDAC3* or hippocampal overexpression of *Nr4a2* were able to ameliorate object location memory impairment in aged mice (Kwapis et al., 2019).

Indeed, Nr4a2 has also been described to play a role in hippocampal neurogenesis and increasing evidence exists supporting that neurogenesis in the sub granular zone of the hippocampus could play a key role in long-term spatial memory. Interestingly, *Nr4a2* is abundantly expressed in adult hippocampal neural precursor cells and it stimulates their proliferation and differentiation both *in vitro* and *in vivo* (Kim et al., 2016). Thus, Nr4a2 could have an important role in hippocampal learning and memory not only by a functional modulation of synaptic plasticity but also by acting through hippocampal neurogenesis.

Although a great deal of evidence has highlighted a role for Nr4a2 in hippocampal-dependent cognitive functions (Table 1), the underlying molecular mechanisms are still poorly understood. *Nr4a2* is induced by neuronal activity and its expression is mainly dependent on CREB/CRTC1 and calcium channels activation (España et al., 2010; Parra-Damas et al., 2014, 2017a,b; Tokuoka et al., 2014). However, knowledge of the molecular pathways involved in Nr4a2 function in synaptic plasticity in the hippocampus is still incomplete. It is known that Nr4a2 transcription factor regulates several genes implicated in hippocampal synaptic plasticity and memory formation (Volpicelli et al., 2007; Hawk et al., 2012; Kim et al., 2020). One example is *Fosl2*, a member of the AP-1 family of transcription factors, a family that is known to be important for memory storage (Fleischmann et al., 2003). Other intriguing Nr4a2 target genes include two receptor protein tyrosine phosphatases, a class of molecules implicated in excitatory synapse formation (Dunah et al., 2005) and the myristoylated alanine-rich C kinase substrate (*MARCKS*), which can modulate memory formation (McNamara et al., 2005). Of particular interest as a candidate target gene by which Nr4a2 influences synaptic plasticity and memory is BDNF, a neurotrophic factor that regulates synaptic plasticity (Zagrebelsky and Korte, 2014) and contributes to the formation and long-term persistence of hippocampus-dependent memories (Bekinschtein et al., 2014; Miranda et al., 2019). Nr4a2 transcriptional activity regulates *BDNF* levels in different systems. Volpicelli et al. (2007) have shown that Nr4a2 regulates *Bdnf* gene expression in rat midbrain neurons and similar results were also reported in cerebellar granule cells (Barneda-Zahonero et al., 2012) and in activated rat microglia overexpressing *Nr4a2* (Chen et al., 2018). Moreover, BDNF was early on implicated in modulating neuronal activity, and its own production and release has been shown to be activity-dependent (Kovács et al., 2007; Parra-Damas et al., 2014). However, relatively little is known about the mechanisms underlying the activity-dependent regulation of *BDNF* expression. Future studies are needed for a fully comprehensive knowledge of the cellular and molecular mechanisms involved in the role of Nr4a2 in hippocampal synaptic plasticity, learning, and memory.

**TABLE 1:** Overview of the overall effects of Nr4a2 in hippocampal synaptic plasticity, learning and memory.

Nr4a2 effects in hippocampal synaptic plasticity, learning and memory	References
**Changes in hippocampal Nr4a2 expression after memory-inducing activities**	
Increase in Nr4a2 mRNA in CA1 and CA3 after acquisition of a spatial food search task in adult rats	Peña de Ortiz et al., 2000
Increase in hippocampal Nr4a2 mRNA and promoter acetylation after contextual fear conditioning in a CREB/CBP-dependent manner in mice	Vecsey et al., 2007 Oliveira et al., 2018 Bridi et al., 2017
Increase in Nr4a2 protein levels with intracranial self-stimulation treatment after the acquisition session of a two-way active avoidance test in rats	Aldavert-Vera et al., 2013
Failure in Nr4a2 increase in the dorsal hippocampus in cognitively impaired aged rats after object recognition memory training	Kwapis et al., 2019
**Impaired hippocampal learning and memory in absence of Nr4a2**	
Nr4a2 siRNA disrupts long-term memory in the object location and object recognition tasks	McNulty et al., 2012
Injection of Nr4a2 antisense oligodeoxynucleotides in the CA3 area impairs long-term memory and reversal learning in an appetitive spatial learning task	Colón-Cesario et al., 2006
Nr4a2 heterozygous mice show reduced ability to form long-term emotional memories in the passive avoidance task	Rojas et al., 2007
The enhancement of location-dependent and novel object recognition long-term memory with HDAC deletion in hippocampal CA1 is specifically abolished by intrahippocampal delivery of Nr4a2 siRNA	McQuown et al., 2011
**Improved hippocampal learning and memory with Nr4a2 overexpression**	
Hippocampal overexpression of Nr4a2 ameliorates the impairment in object location memory in aged mice	Kwapis et al., 2019
**Nr4a2 regulates hippocampal neurogenesis**	
Nr4a2 stimulates the proliferation and differentiation of adult hippocampal neural precursor cells both *in vitro* and *in vivo*	Kim et al., 2016
**Nr4a2 regulates genes involved in hippocampal synaptic plasticity and memory formation**	
Fosl2	Fleischmann et al., 2003
Receptor protein tyrosine phosphatases	Dunah et al., 2005
Myristoylated alanine-risch C kinase substrate (MARCKS)	McNamara et al., 2005
BDNF	
In rat midbrain neurons	Volpicelli et al., 2007
In cerebellar granule cells	Barneda-Zahonero et al., 2012
In rat microglia	Chen et al., 2018

## Is Nr4a2 a Synaptonuclear Protein Messenger?

Long-term memory involves persistent changes in synaptic proteome and structure that require the regulation of gene transcription (Alberini, 2009; Heo et al., 2018). Transcription factors involved in learning and memory will translocate in and out the nucleus in response to the signaling cascades activated at synapses (Ch’ng and Martin, 2011). Several pathways, typically requiring calcium signaling, have been described to connect synaptic inputs to gene transcription (Hagenston and Bading, 2011). Activation of different protein kinases regulates the phosphorylated state of transcription factors and their cytosol-nuclear localization (Klann and Dever, 2004). However, the view that activity-dependent transcriptional regulation is based on post-translational modification of transcription factors located in the cytosol or nucleus is changing. Several transcription modulators involved in learning and memory, such as CRTCs, NFκB or HDAC4, have been reported to be located at synapses and to be transported to nucleus upon synapse stimulation (Uchida and Shumyatsky, 2018). CRTC1 has been found in the postsynaptic density (PSD) subfraction obtained from adult mouse brain and in excitatory synapses from hippocampal cultures. It translocates to nucleus in response to localized synaptic activity or induction of LTP (Ch’ng Toh et al., 2012). Histone deacetylase 4 (HDAC4) shuttles between the cytosol and the nucleus in response to neuronal activity. Although HDAC4 is mainly located in the neuronal cytoplasm with a more variable nuclear presence, immunohistochemistry studies have shown a strong PSD immunoreactivity in dendritic spines (Darcy et al., 2010). Another transcriptional modulator with a synaptic localization is NFκB (Kaltschmidt et al., 1993; Meffert et al., 2003), which is activated upon depolarization (Meffert et al., 2003). Other transcriptional modulators are known to be present at synapses, such as Arc and ATF2 (Lai et al., 2008; Shepherd and Bear, 2011) and it is likely that the list of transcriptional regulators that participate in synapse to nucleus signaling will increase in the future.

**FIGURE 2:**
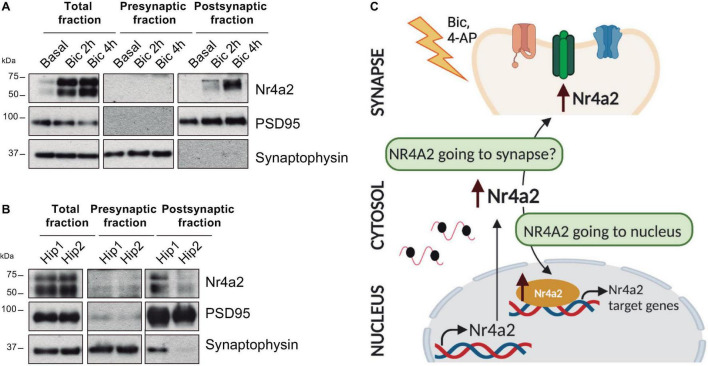
Nr4a2 transcription factor is present at the postsynaptic density both in mature hippocampal-cultured neurons and the adult mouse hippocampus. **(A)** Synaptoneurosomes from mature hippocampal-cultured neurons after bicuculline (bic; 50 μM) treatment. **(B)** Synaptoneurosomes of adult mouse hippocampus (hip). All figures correspond to representative images from Western blotting analysis. *n* ≥ 3. **(C)** Schematic model of Nr4a2 mobilization to nucleus and postsynaptic density fractions after neuronal activity.

Nr4a2 contains a bipartite nuclear localization signal (NLS) within its DBD and three leucine-rich nuclear export signals (NES) in its LBD. Together, these signals regulate Nr4a2 shuttling in and out of the nucleus (García-Yagüe et al., 2013). Notably, data from our laboratory support the presence of Nr4a2 in the postsynaptic density fraction of mature hippocampal neuronal cultures treated with the GABA-A receptor antagonist bicuculline (Figure 2A). Moreover, Nr4a2 was also detected in the postsynaptic density fraction of synaptoneurosomes obtained from adult mouse hippocampus (Figure 2B). These data open the possibility that Nr4a2 could also be considered as a synaptically localized transcriptional regulator that will be transported, by an unknown mechanism, upon synaptic stimulation (Figure 2C). Further future work is needed to confirm the presence of Nr4a2 in the synapse and the importance of this subcellular localization in the already described role of Nr4a2 in hippocampal synaptic plasticity and memory formation.

## Nr4a2 in Cognitive and Neuropsychiatric Brain Disorders

**FIGURE 3:**
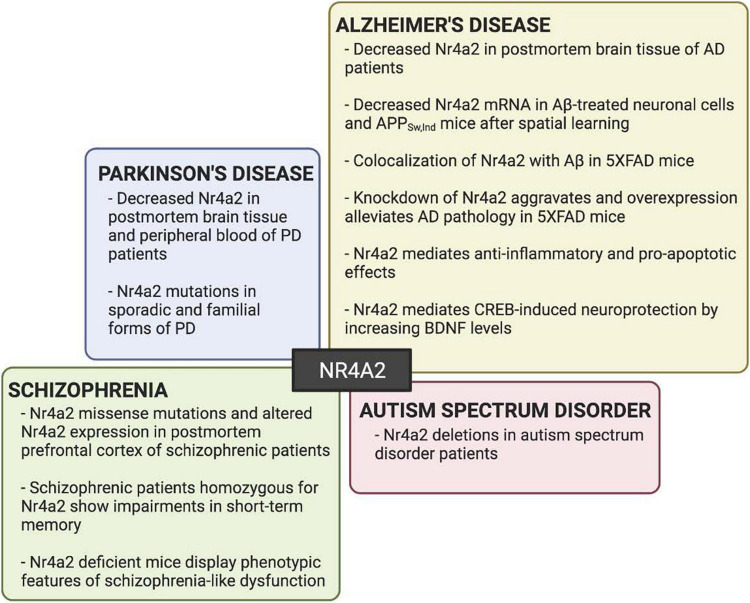
Nr4a2 involvement in different cognitive and neuropsychiatric brain disorders. Schematic representation of Nr4a2 functions and/or alterations in neurodegenerative disorders (Parkinson’s disease, Alzheimer’s disease), schizophrenia and autism spectrum disorder.

Parkinson’s disease (PD), which results from the degeneration of midbrain dopaminergic neurons, was the first brain pathology that was related to Nr4a2. Early reports showed that *Nr4a2* expression was diminished in both aged and PD brains (Chu et al., 2002). Later, *Nr4a2* gene expression was found reduced not only in the postmortem brain tissue, but also in the peripheral blood of PD patients (Le et al., 2008; Montarolo et al., 2016). Moreover, *Nr4a2* mutations or polymorphisms were detected in sporadic and familial forms of PD (Xu et al., 2002; Le et al., 2003; Zheng et al., 2003; Grimes et al., 2006; Sleiman et al., 2009). Besides PD, evidence supports a role for Nr4a2 in the pathogenesis of different CNS disorders (Figure 3). In recent years, much attention has been directed to Nr4a2 function in hippocampal synaptic plasticity, learning, and memory, addressing much interest to the role of Nr4a2 in brain pathologies that course with cognitive or intellectual disabilities. One of the first brain diseases, besides PD, that was related to Nr4a2 was Alzheimer’s disease (AD).

The potential of some nuclear receptors to serve as therapeutic targets in disorders of cognition including AD has been reported (Mandrekar-Colucci and Landreth, 2011; Skerrett et al., 2014). CREB is one of the transcription factors most extensively studied in AD, being disrupted by oligomeric forms of amyloid-β (oAβ) and leading to synaptic plasticity and memory deficits in AD (Vitolo et al., 2002; Saura and Valero, 2011). Moreover, CREB transcription factor represents the crossroad of different synapse-to-nucleus pathways associated with changes in gene expression that underlie memory decline in AD (Marcello et al., 2018). Pioneer genome-wide transcriptome profile analyses of AD transgenic mice hippocampus revealed deregulation of a transcriptional program dependent on CREB/CRTC1. Specifically, APP_*Sw,Ind*_ mice at 6 months of age showed down-regulation of diverse genes compared with wild type mice after spatial training. These genes include *Nr4a2* (España et al., 2010; Parra-Damas et al., 2014). Indeed, a specific decrease of Nr4a2 levels in Alzheimer’s pathology has been observed. Nr4a2 mRNA levels were found diminished in Aβ_1–42_-treated neuronal cells (Terzioglu-Usak et al., 2017) and in AD mouse models. A reduction in Nr4a2 mRNA was also seen in APP_*Sw,Ind*_ mice when initial hippocampal-dependent spatial memory deficits appeared (España et al., 2010; Parra-Damas et al., 2014). A similar decrease was also reported in postmortem brains of human AD patients, specifically in the frontal cortex, and the hippocampal formation (Parra-Damas et al., 2014; Moon et al., 2019). A role of Nr4a2 in AD pathology was further supported by the observation that Nr4a2 protein was prominently expressed in brain areas with Aβ accumulation in the 5XFAD mouse model of AD and, notably, it was highly co-expressed with Aβ at mice ages mimicking early stages of the disease (Oakley et al., 2006). In addition, the number of Nr4a2-expressing cells significantly declined in the 5XFAD mouse in an age-dependent manner, accompanied by increased plaque deposition, suggesting a possible causal-effect relation between Nr4a2 levels and AD progression (Moon et al., 2015). Moreover, in a recent study performed by the same laboratory (Moon et al., 2019), they found that knockdown of Nr4a2 significantly aggravated AD pathology while its overexpression alleviated it, including a decrease in Aβ accumulation and neurodegeneration. Similar observations were obtained in 5XFAD mice treated with Nr4a2 activators.

It is noteworthy to mention that the functional roles of Nr4a2 in AD could not be limited to its implication in synaptic function, but also be extended to its involvement in the inflammatory and neuronal death processes associated to AD. For example, it was described that inflammatory stimulus up-regulated *Nr4a2* expression in microglia (Fan et al., 2009). This up-regulation could trigger a compensatory response through modulation of the NFκB action. Nr4a2 binds to target inflammatory gene promoters thought association with NFκB and the CoREST repressor complex down-regulating the expression of several pro-inflammatory genes in microglia and astrocytes (Saijo et al., 2009). Furthermore, Nr4a2 neuroprotective effects are not limited to dopaminergic neurons. In glutamatergic neurons, Nr4a2 also mediates CREB-induced neuroprotection in response to stress by increasing BDNF levels in cerebellar granule cells (Barneda-Zahonero et al., 2012) or up-regulating an anti-apoptotic gene program in hippocampal neurons (Volakakis et al., 2010). Altogether, this anti-inflammatory and pro-survival role in brain cells could also be involved in the reduction of neuroinflammation and neuronal death seen in AD mice models (Moon et al., 2019).

Some reports have also associated Nr4a2 with schizophrenia. This psychiatric disease is believed to result from subtle disturbances during brain development that lead to improper function of synaptic transmission and plasticity of several neurotransmitter systems (Birnbaum and Weinberger, 2017). A consistent neuropathological observation in postmortem brains of schizophrenic patients is the reduction in the density of small dendritic spines (MacDonald et al., 2017), which are more plastic than bigger spines and, therefore, they are related to learning and memory processes (Matsuzaki et al., 2001; Trachtenberg et al., 2002). Missense mutations in the third exon of *Nr4a2* (Buervenich et al., 2000) or altered expression of *Nr4a2* in postmortem prefrontal cortex were detected in schizophrenic patients (Guillozet-Bongaarts et al., 2014; Corley et al., 2016). Moreover, schizophrenic patients homozygous for variants of the *Nr4a2* promoter showed impairments in short-term memory assessed by the Backward Digit Span task (Ruiz-Sánchez et al., 2021). A putative relationship between Nr4a2 and schizophrenia was further supported by studies with rodents. *Nr4a2* heterozygous mice displayed a pleiad of symptoms and behaviors related to schizophrenia (Rojas et al., 2007; Vuillermot et al., 2011). These mice showed hyperactivity in novel environments and were deficient in the passive avoidance learning task. Moreover, a depression-like profile was also noticed in the forced-swimming test (Rojas et al., 2007). The behavioral profile of *Nr4a2* heterozygous mice regarding schizophrenia-relevant phenotypes was further extended by Vuillermot et al. (2011). They reported that *Nr4a2*-deficient male mice show sensorimotor gating deficits consistent with an alteration in the glutamatergic system. This deficit in sensorimotor gating has been consistently reported in schizophrenia patients (Swerdlow and Geyer, 1998; Braff et al., 2001). Altogether, these findings indicate that *Nr4a2*-deficient mice display some phenotypic characteristics of schizophrenia-like dysfunction.

The presence of *Nr4a2* deletions in patients with autism spectrum disorder has also related Nr4a2 with intellectual disability. In some reports, the deletion affected *Nr4a2* and *GPD2* genes and caused intellectual disability and language impairment (Barge-Schaapveld et al., 2013; Leppa Virpi et al., 2016). However, other studies reported an individual with mild intellectual disability and prominent speech and language impairment that presented a deletion encompassing only Nr4a2 (Reuter et al., 2017; Lévy et al., 2018). These studies provide further evidence for *Nr4a2* haploinsufficiency being associated with intellectual disability and autism spectrum disorder.

All these studies clearly show that the involvement of Nr4a2 in brain diseases expands beyond PD and point out this transcription factor as a promising candidate for the development of novel therapeutic approaches. Future work should provide solid grounds to determine the precise contribution of Nr4a2 to each brain disorder.

## Therapeutic Potential of Nr4a2 Modulation

As discussed above, until recently, it was considered that Nr4a family members were orphan nuclear receptors since their LBD seemed to avoid the binding of ligands due to the tight packing of bulky hydrophobic side chain residues (Wang et al., 2003). However, during the last decade, several nuclear magnetic resonance (NMR) studies of the isolated Nr4a2-LBD have suggested that several synthetic small molecules could bind Nr4a2 in canonical and non-canonical binding pockets (Kim et al., 2015; de Vera et al., 2019). Moreover, recent studies have shown that the Nr4a2-LBD could be highly dynamic (de Vera et al., 2019) allowing the binding of putative endogenous ligands such as docosahexaenoic acid, prostaglandin E1 and the dopamine metabolite 5,6-dihydroxyndole (de Vera et al., 2016; Bruning et al., 2019; Rajan et al., 2020). At present, it is still unknown the physiological importance of these putative endogenous ligands on Nr4a2-mediated role in the CNS.

The identification of synthetic small molecules that could modulate Nr4a2 transcriptional activity opens the possibility to provide target-based therapies for brain diseases related to Nr4a2. Among the panoply of Nr4a2 modulators identified so far (Table 2), of special interest are the antimalarial drugs amodiaquine (AQ) and chloroquine (CQ), which increase the transcriptional function of Nr4a2 interacting with its LBD through direct physical binding. These drugs were identified as Nr4a2 agonists after screening a chemical library composed by Food and Drug Administration (FDA)-approved drugs in a cell-based luciferase assay system (Kim et al., 2015). NMR spectroscopy suggested the binding of both drugs to the Nr4a2-LBD. Accordingly, AQ and CQ were able to increase the transcriptional activity of Nr4a2 in human neuroblastoma cell lines (Kim et al., 2015). In addition, in preclinical studies, these compounds meaningfully improved behavioral deficits in rat models of PD without any noticeable sign of dyskinesia-like behavior (Kim et al., 2015). Autophagic-lysosomal blockade was also reported as one of their functions (Qiao et al., 2013). Recently, AQ has also been found to enhance cognitive functions by increasing adult hippocampal neurogenesis (Kim et al., 2016). Importantly, 5XFAD mouse model of AD treated with the Nr4a2 agonist AQ showed robust reduction in typical AD features including deposition of Aβ plaques, neuronal loss, microgliosis and impairment of adult hippocampal neurogenesis, leading to significant improvement of cognitive functions in the Y-maze, which is a behavioral paradigm widely accepted to evaluate spatial working memory. The same study also showed for the first time that AQ treatment significantly inhibited γ-secretase activity and enhanced degradation of Aβ via up-regulation of insulin-degrading enzyme, an Aβ-degrading protease (Moon et al., 2019).

**TABLE 2:** Features of synthetic and endogenous Nr4a2 agonists.

Compound	Target	Model	Outcomes	References
**Endogenous ligands**				
Docosahexaenoic acid	Nr4a2-LBD	HEK-293T, MN9D cells	Enhanced Nr4a2 transcriptional activity	de Vera et al., 2016
Prostaglandins (PGE1 and PGA1)	Nr4a2-LBD	SK-N-BE(2)C, MN9D and N27-A cells	Enhanced Nr4a2 transcriptional activity, neuroprotection, improved behavioral deficits in a PD mouse model	Rajan et al., 2020
5,6-Dihydroxyindole (DHI)	Nr4a2-LBD	JEG3 cells	Enhanced Nr4a2 transcriptional activity	Bruning et al., 2019
**Synthetic ligands**				
**Antimalarial drugs**				
Chloroquine Amodiaquine	Putative LBD residues	DA neurons, PC12 cells 6-OHDA lesioned rats (PD model) A375 melanoma cells C57BL/6 mice 5XFAD mice (AD model)	Increased expression of DA genes; anti-inflammatory responses, neuroprotection Improved behavioral deficits Autophagic-lysosomal blockade Enhanced adult hippocampal neurogenesis, improved cognition Inhibited Aβ-mediated pathology; improved cognitive functions	Kim et al., 2015 Qiao et al., 2013 Kim et al., 2016 Moon et al., 2019
**Bicyclic compounds**				
6-Mercaptopurine	AF-1 domain	CV-1 cells Microglia	Activation of both Nr4a2 and Nr4a3 Anti-inflammatory responses	Ordentlich et al., 2003 Huang et al., 2016
Isoxazolopyridone-based compounds (IP7e)	Putative LBD residues	OF1 mice (PD model) Multiple sclerosis models	Increased DA levels in the SN and striatum Inhibited expression of NFκB (attenuated inflammation and neurodegeneration), improved rotarod	Hintermann et al., 2007 Montarolo et al., 2014
SA00025	Unknown	6-OHDA lesioned rats (PD model)	Increased expression of DA genes; anti-inflammatory response and neuroprotection	Smith et al., 2015
DIM-C analogs	Both N- and C-terminus Nr4a2; direct binding not supported	BV-2 cells MPTP lesioned rats (PD model) C57BL/6 mice C57BL/6 mice	Suppressed NFκB-induced genes Increased expression of DA genes; anti-inflammatory response and neuroprotection Enhanced long-term spatial memory in young mice, rescued memory deficits in aged mice Enhanced hippocampal LTP and long-term contextual fear memory	De Miranda et al., 2015 Chatterjee et al., 2020 Bridi et al., 2017

Mercaptopurine was the first Nr4a2 agonist to be identified (Ordentlich et al., 2003). However, although it has been reported that produces anti-inflammatory responses in microglia by attenuation of TNF-α production (Huang et al., 2016), it is not considered a specific Nr4a2 agonist (Wansa et al., 2003; Huang et al., 2016). Other bicyclic compounds described to enhance Nr4a2 transcriptional activity as homodimer or heterodimer are isoxazolopyridinone-based compounds developed by Novartis AG (Hintermann et al., 2007). Among them, isoxazolo-pyridinone 7e (IP7e) has an excellent oral bioavailability and a fast and extensive brain uptake and has been reported to ameliorate neuroinflammation and neurodegeneration in an experimental model of autoimmune encephalomyelitis (Montarolo et al., 2014). However, a recent study suggests that IP7e affects general transcription via Nr4a2-independent mechanisms (Munoz-Tello et al., 2020) seeding doubts about its role as a specific Nr4a2 agonist. Another bicyclic compound, SA00025, a 2-aryl-6-phenylimidazol[1,2-α]pyridine derivative produced by Sanofi Aventis, was reported to exhibit partial neuroprotective effect in PD mouse models (Smith et al., 2015). Although ligand screening shows an EC_50_ on the low nanomolar range (Jang et al., 2021), no data have shown its direct binding to Nr4a2.

Another group of small molecules that have been identified to specifically activate members of the Nr4a family of transcription factors are the paraphenyl substituted diindolylmethane analogs or C-DIM (Li et al., 2012). Some of the C-DIM compounds, as C-DIM5 and C-DIM8, have a greater specificity for Nr4a1 than for Nr4a2. By contrast, C-DIM12 has higher affinity for Nr4a2 and it is able to exert a potent anti-inflammatory and neuroprotective role in a mouse model of PD (De Miranda et al., 2015). Moreover, it enhances long-term spatial memory in young mice and rescues memory deficits in aged mice (Chatterjee et al., 2020). In summary, the possibility that Nr4a2 could be drug-targeted with several small synthetic molecules opens an attractive and promising future for developing therapeutic strategies that include Nr4a2 as a druggable target.

## Concluding Remarks and Future Research

During the last decade, many reports have supported a major role for the Nr4a family of nuclear receptors in hippocampal synaptic plasticity and memory processes. The role of Nr4a2, a member of this family, has received special attention. Several data suggest that Nr4a2 activation is necessary for hippocampal LTP and hippocampal-mediated learning tasks. However, many questions remain unanswered. It is still unknown which are the signaling pathways that link synaptic activity to *Nr4a2* induction. We don’t know yet how Nr4a2 activation is enhancing hippocampal plasticity and learning. Which are the genes activated or repressed by Nr4a2? Does Nr4a2 act on excitatory and/or inhibitory synapses? Is Nr4a2 modulating the pre and/or postsynaptic function? Which impact has glial Nr4a2 in synaptic plasticity? Work during the following years will confirm whether Nr4a2 is present at the synapse and could act as a synaptonuclear messenger and how its subcellular compartmentalization is modulated.

Future studies should improve our knowledge of the role of Nr4a2 in the cognitive impairment associated to several brain pathologies. More pre-clinical studies are needed to confirm the importance of Nr4a2 activation in the hippocampus to overcome the learning and memory deficits observed in animal models of AD. It is necessary to extend the studies on the role of Nr4a2 in AD beyond the 5XFAD mouse model. Also, an extended behavioral characterization of learning and memory in these animals is needed. It would be also relevant to know whether Nr4a2 activation has any impact on Aβ plaques and neurofibrillary tangles, which are the neurohistological hallmarks of AD. Moreover, the relationship between Nr4a2 and some psychiatric diseases has been already established. Thus, it would be also important to address the impact of Nr4a2 on behavioral and psychological symptoms with new studies in animals’ models for these diseases such as schizophrenia or autism spectrum disorder.

All these studies will benefit from the already known Nr4a2 ligands that allow us to consider Nr4a2 as a druggable target. However, to provide future target-based therapeutic interventions, more work should be done to modify existing chemical structures to increase the specificity of Nr4a2 agonists, increase their blood-brain barrier permeability and improve their pharmacokinetic characteristics.

Finally, another important question needs to be answered during the next decade. Is Nr4a2 an orphan nuclear receptor? Although this question has been considered affirmative until now, recent studies suggest that endogenous ligands could indeed bind to Nr4a2-LBD. Future confirmation of the existence of such endogenous ligands will open an important scenario, since additional mechanisms, besides protein levels, could regulate Nr4a2 activity.

## Author Contributions

JC-S, AM-M, and JR-Á conceived the idea for this review and reviewed the literature. JC-S wrote the first draft of the manuscript and created the figures. JR-Á wrote the final draft. JC-S and AM-M revised the manuscript. All authors contributed to the article and approved the submitted version.

## Conflict of Interest

The authors declare that the research was conducted in the absence of any commercial or financial relationships that could be construed as a potential conflict of interest.

## Publisher’s Note

All claims expressed in this article are solely those of the authors and do not necessarily represent those of their affiliated organizations, or those of the publisher, the editors and the reviewers. Any product that may be evaluated in this article, or claim that may be made by its manufacturer, is not guaranteed or endorsed by the publisher.
